# Effect of the gut microbiome and inflammation-related proteins on oral leukoplakia: a Mendelian randomization study and mediation analysis

**DOI:** 10.3389/fonc.2024.1443123

**Published:** 2024-09-25

**Authors:** Junlong Da, Yinting Ren, Shiwei Liu, Nanyan Wang, Lei Wang, Zhifeng Fu, Yongtang Liang, Yu Pan, Jin Li, Jufeng Chen

**Affiliations:** ^1^ Department of Stomatology Center, The First People’s Hospital of Foshan, Foshan, China; ^2^ Department of Stomatology, Foshan Hospital of Traditional Chinese Medicine, Foshan, China

**Keywords:** gut microbiota, oral leukoplakia, inflammation-related proteins, Mendelian randomization, mediation analysis

## Abstract

**Background:**

Oral leukoplakia (OL) is the most common potentially malignant disease of the oral cavity. In recent years, studies have identified a correlation between the gut microbiota (GM) and oral cancer, in addition, inflammation-related proteins have been reported to play an important role in the development of OL. However, the causal relationship between gut microbiota and OL, as well as whether cytokines play a mediating role, remain unclear.

**Methods:**

In this Mendelian randomization (MR) study, the genome-wide association studies (GWAS) (n=18340) of the MiBioGen consortium microbiome was used as exposure data. Genetic variation data related to OL were extracted from the Finngen R9 project (513 cases of OL and 411668 controls). The 91 inflammation-related proteins obtained in the literature serve as potential mediators. Two-sample Mendelian randomization analysis was applied to infer causality using Inverse Variance Weighted (IVW), MR Egger, weighted media, simple mode and weighted mode method. Subsequently, sensitivity analysis was conducted to ensure the robustness of the MR results. In addition, we conducted reverse MR analysis to alleviate reverse causality. Finally, we used mediation analysis to determine the pathway mediated by inflammation-related proteins from the gut microbiota to OL.

**Results:**

The five bacterial taxa in the gut microbiota indicate a potential causal relationship with the development of OL. Notably, *family Clostridiaceae1* was negatively correlated with the risk of OL development, while *genus Dorea*, *genus Ruminococcus1*, *genus Senegalimasilia* and *genus Veillonella* were positively associated with the risk of OL development. In addition, this study identified a potential causal relationship between interleukin-10 receptor subunit alpha (IL-10RA), interleukin-18 receptor 1(IL18-R1) and the occurrence of OL. In addition, intermediary analysis indicates that IL18-R1 mediated the pathway between the gut microbiota *genus Senegalimasilia* and OL.

**Conclusions:**

In summary, our research emphasize the complex relationship between gut microbiota, inflammation-related proteins and OL. The identified associations and mediating effects provide new insights into potential therapeutic approaches for targeting the gut microbiota in the management of OL, and contribute to its prevention, diagnosis and treatment.

## 1 Introduction

Oral potentially malignant disease (OPMD) is characterized by tissue changes that are more likely to transform into oral cancer than normal tissue (1). Oral squamous cell carcinoma (OSCC) is the most common type of oral cancer, accounting for more than 90% of oral malignancies (2). According to the World Health Organization, oral leukoplakia (OL), defined as “a suspected risky white plaque that excludes other known diseases but does not increase the risk of cancer,” is the most common OPMD (1, 3, 4),with an estimated global prevalence of between 1.5 and 4.1% (5). Current research indicates that the pooled proportion of malignant transformation (MT) from OL to OSCC is 9.8% (6). Until now, there is no effective treatment to prevent MT in OL (7). Regular follow-up may help in the early detection of potentially malignant transformation, but its cost-effectiveness and best practices still need to be further investigated.

The gut microbiota is a vast microbial community that lives in the human gut and is considered the largest microbial reservoir in the human body, including bacteria, archaea, viruses, fungi, protozoa, and parasites (8, 9). It is especially noteworthy that the community contains over 10 trillion different microorganisms, accounting for approximately 90% of the total human microbial population (10). The gut microbiota plays an important role in many physiological and health-related processes in humans, including immune system development, vitamin production and nutrient absorption, through interactions with the host (11). The oral microbiota is the second largest microbiota in the human body after the intestinal microbiota. The oral microbiota is composed of over 700 types of bacteria, as well as fungi, viruses, and protozoa (12). Current research has found that OL is associated with a decrease in *Firmicutes* levels in the oral microbiota, including an increase in the abundance of anaerobic bacteria such as *Fusobacterium nucleatum, Prevotella intermedia* and *Porphyromonas gingivalis*, which are considered periodontal pathogens (13). The consumption of *Firmicutes* is often reported as precancerous and malignant lesions (14, 15). Considering that the oral mucosa and gastrointestinal mucosa are physically connected, and existing research has demonstrated the interrelationship between the oral and intestinal microbiota (16), it is reasonable to hypothesize that gut flora may influence the development of OL in certain form.

Inflammation is a defensive response of the body to external stimuli and an important component of the immune system, playing an indispensable role in resisting damage and infection. However, when inflammation loses control or persists for a long time and becomes chronic, it can lead to cell damage, tissue destruction, cancer, and even death (17, 18). Previous studies have supported the role of inflammatory mediators in the pathogenesis of OL to OSCC (19). The results of the study showed that salivary concentrations of interleukin-6 (IL-6) were significantly higher in patients with clinically proven OL compared to healthy individuals. In addition, elevated levels of tumor necrosis factor alpha (TNF-α) and IL-8 were found in OL samples (20, 21). However, the exact role of these cytokines in OL is still unclear. The current research has established a connection between gut microbiota and inflammatory mediators. The changes in gut microbiota are usually related to abnormal immune responses and dysregulation of inflammatory cytokine production (22, 23). Therefore, it is reasonable to speculate that there may be a relationship between gut microbiota, inflammatory mediators and OL. Our research aims to elucidate these potential correlations and identify specific inflammatory cytokines that can serve as valuable tools for early diagnosis and potential clinical treatment.

Mendelian randomization (MR) is a genetic epidemiological method for determining causal relationships between exposures and outcomes by using genetic variation as an instrumental variable (24, 25). Since genetic variants detected in fertilized eggs are identified at the time of conception, MR is less subject to traditional environmental confounding variables and reverse causation than observational studies (26, 27). MR has become a key epidemiological method for inferring causal relationships (28). Therefore, based on the significant reliability of previous MR studies in determining the etiology, in this study, we aim to explore the correlation between gut microbiota, inflammatory mediators and OL through MR analysis of the aggregated data from publicly available genome-wide association studies (GWAS). These analyses aim to dissect the intricate correlations between these variables and provide valuable insights into their causal relationships.

## 2 Materials and methods

### 2.1 Study design

**Figure 1:**
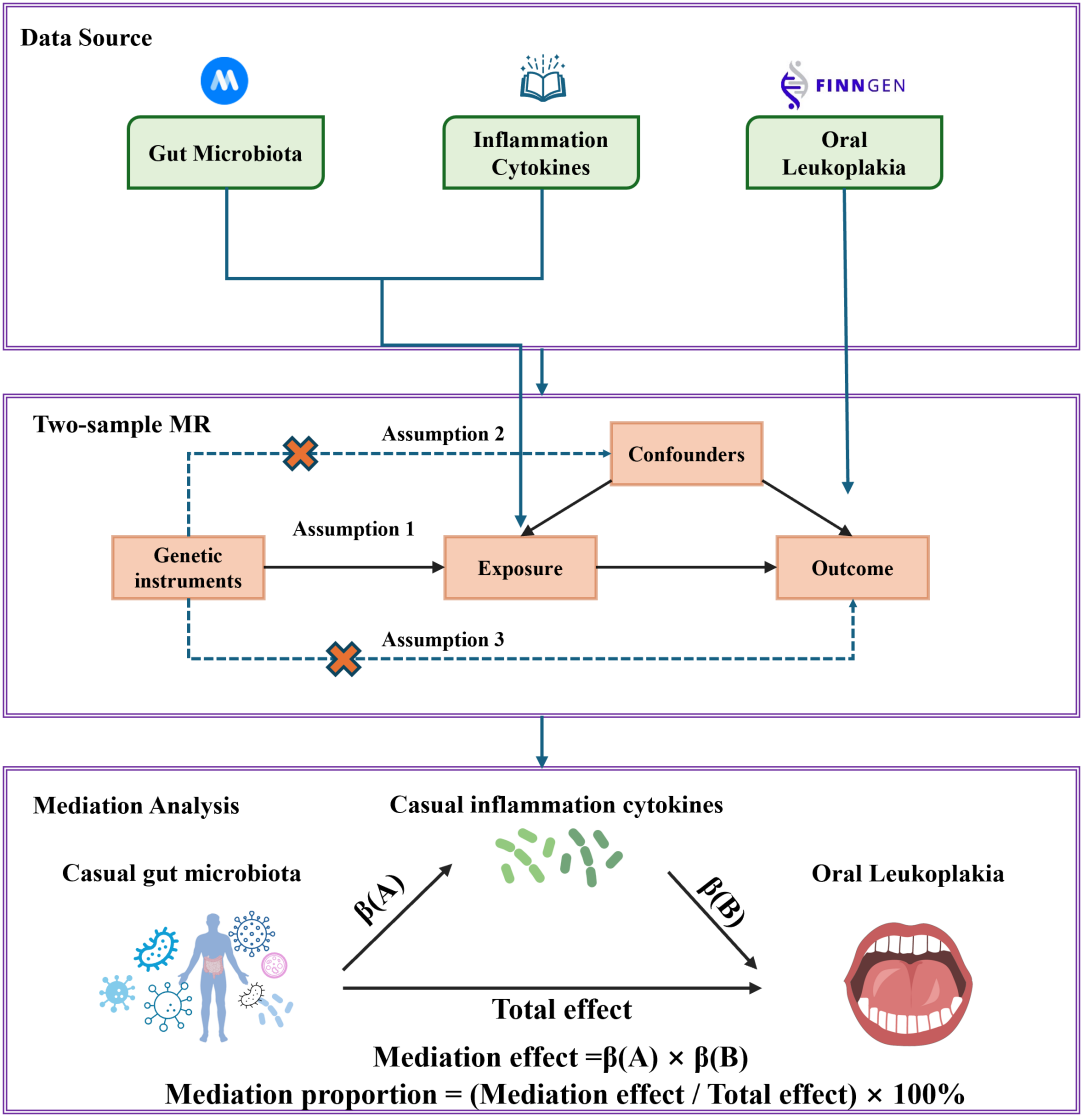
Study design and flow chart.

The research flowchart is shown in **Figure 1**. Firstly, we obtained summary statistics from published GWAS that included characteristics of the gut microbiota, inflammation-related proteins and OL. Secondly, two-sample MR analysis was used to assess causal relationships between gut microbiota, inflammation-related proteins and OL. Finally, two-step and multivariate MR (MVMR) analyses were used to determine the mediating role of inflammation-related proteins on the relationship between gut microbiota and OL. In order to obtain unbiased causal effects, MR analyses should adhere to the following three hypotheses: (1) Correlation hypothesis: instrumental variables (IVs) are strongly associated with exposure. (2) Independence hypothesis: IVs must be independent of potential confounders. (3) Exclusion limitation hypothesis: IVs affect the outcome of interest only through association with the exposure of interest, and there is no other way to play a role (29). This study followed the reporting guidelines of STROBE-MR (25). As this study is based on publicly available data, no additional ethical approval or consent is required.

### 2.2 Data source

Gut microbiota GWAS data were obtained from the MiBioGen study (30), which includes genome-wide genotypes of 18340 individuals (24 cohorts) from 11 countries, as well as fecal microbiota data obtained through 16S rRNA gene sequencing. The GWAS summary data includes 211 taxa of gut microbiota (131 genera, 35 families, 20 orders, 16 classes, 9 phyla), which is the most extensive multi group study on gut microbiota to date. The consortium has carefully adjusted for variables such as sex, age, and genetic principal components (PCs). They also included alpha diversity indices and technical covariates like DNA isolation methods and genotyping platforms. Quality control procedures, including minor allele frequency (MAF) thresholds and outlier removal, were also applied (31).

The GWAS summary data for oral leukoplakia is sourced from the 10th version of FinnGen Consortium(https://r10.risteys.finngen.fi/endpoints/K11_ORAL_LEUCOPLACIA) (32), which included 513 individuals diagnosed with oral leukoplakia and 411668 control subjects (FinnGen data field: K11_ORAL_LEUCOPLACIA). To maintain uniformity, all study participants are of European descent.

We obtained updated GWAS summary statistics for 91 inflammation-related proteins (the GWAS catalog ranges from GCST0001391 to GCST0002121) from the study by Zhao et al. (33), which recruited 14,824 participants mainly from Europe. Detailed information on data processing and complete data sources are available in the original literature.

### 2.3 Instrumental variables selection

Firstly, we utilized single nucleotide polymorphisms (SNPs) as IVs to investigate the causal relationship between exposure and outcomes at the genetic level. To ensure the accuracy and effectiveness of the causal relationship between gut microbiota and oral leukoplakia, we selected SNPs (P<1 × 10^-5^) that are significantly correlated with gut microbiota (34). In order to maximize the available instrumental variables for each inflammation-related proteins, we selected SNPs with a P-value of 5 × 10^-6^ as the threshold to obtain more comprehensive results and explore more potential relationships between OL and inflammation-related proteins (35). Then we excluded SNPs with linkage disequilibrium (LD) in the analysis. The selected SNPs with LD should meet the conditions of r^2^<0.001 and distance>10,000 kb with gut microbiota to minimize the linkage imbalance effect that violates random allele allocation. In addition, SNPs exhibiting palindromic or fuzzy features are systematically excluded from MR analysis. (Palindrome SNPs are SNPs with A/T or G/C alleles.) The strength of IVs is evaluated by calculating the F-statistic (Equation 1). Among them, N represents the sample size, K represents the number of instruments, R2 is used to represent the exposure variance explained by the selected SNP. If the corresponding F-statistic is less than 10, IVs are considered weak IVs and then excluded; On the contrary, F-statistics above 10 indicate that IVs have sufficient strength and are considered to have no significant weak instrument bias.


(1) $$\text{F}-\text{statistic}=\frac{\text{n}-\text{k}-1}{\text{k}}\times \frac{{\text{R}}^{2}}{1-{\text{R}}^{2}}$$

### 2.4 MR analysis and Mediation analysis

Causal relationships between gut microbiota, inflammation-related proteins, and oral leukoplakia were assessed using MR methods. The Wald ratio (WR) method was used to estimate the effect of individual IVs on causal estimates (36). For exposures that included multiple IVs, causality was inferred using the inverse variance weighted (IVW), MR Egger, weighted median, simple mode, and weighted mode methods. Of these, the IVW, which weights the inverse variance of the causal effects of different genetic variants on a trait and then combines the weighted effect estimates, was used as the primary method, while the other methods were used as supplements. We aimed to minimize bias and obtain reliable estimates that could alter the causal relationship between exposure and the outcome of interest.

Mediation analysis aims to evaluate the pathways from exposure to outcomes through mediation, which helps to explore the potential mechanisms by which exposure affects outcomes. Firstly, two sample MR methods were used to evaluate the causal relationship between gut microbiota and inflammation-related proteins to obtain β(A). Next, we determined the direct impact of gut microbiota on oral leukoplakia through MVMR to obtain β(B). We calculate the mediating effect by multiplying β (A) by β (B). To quantify the degree of mediation, we obtained the total impact of gut microbiota on obesity in the previous two-sample MR. We divided the mediating effect by the total effect of gut microbiota in OL. Two steps of MR were significant (P<0.05) and overlapping gut microbiota were considered as mediators.

### 2.5 Sensitivity analysis

To evaluate the robustness of causal relationships, we conducted sensitivity analysis. Sensitivity analysis includes multiple validity analysis, heterogeneity analysis and leave-one-out analysis. MR Egger regression analysis is used to evaluate the potential pleiotropic effects of SNPs used as IVs, The MR Egger intercept is used to indicate horizontal pleiotropy. Excessive horizontal pleiotropy indicates that the analysis violates the basic assumptions of MR analysis, making the results less reliable. The Cochran Q-test is used to measure the heterogeneity of IVs (37). In addition, a “leave-one-out” analysis was conducted to determine the potential bias effect of a single SNP on MR analysis, and this impact was re estimated by sequentially removing one SNP at a time. In order to clearly and concisely visualize the results of the MR method, the “TwoSampleMR” software package was used (38). All statistical analyses in this study were conducted using the R package in R (v4.2.1) statistical software.

### 2.6 Reverse MR analysis

To investigate whether oral leukoplakia has a causal effect on the identified significant gut microbiota (P_IVW_<0.05), we performed reverse MR analysis using SNPs associated with OL as IVs, and identified gut pathogens as the outcomes. The reverse MR analysis program is similar to the program used for MR analysis to elucidate the directional causal relationship between exposure and outcome.

## 3 Results

### 3.1 Causal effects of gut microbiota on OL

**Figure 2:**
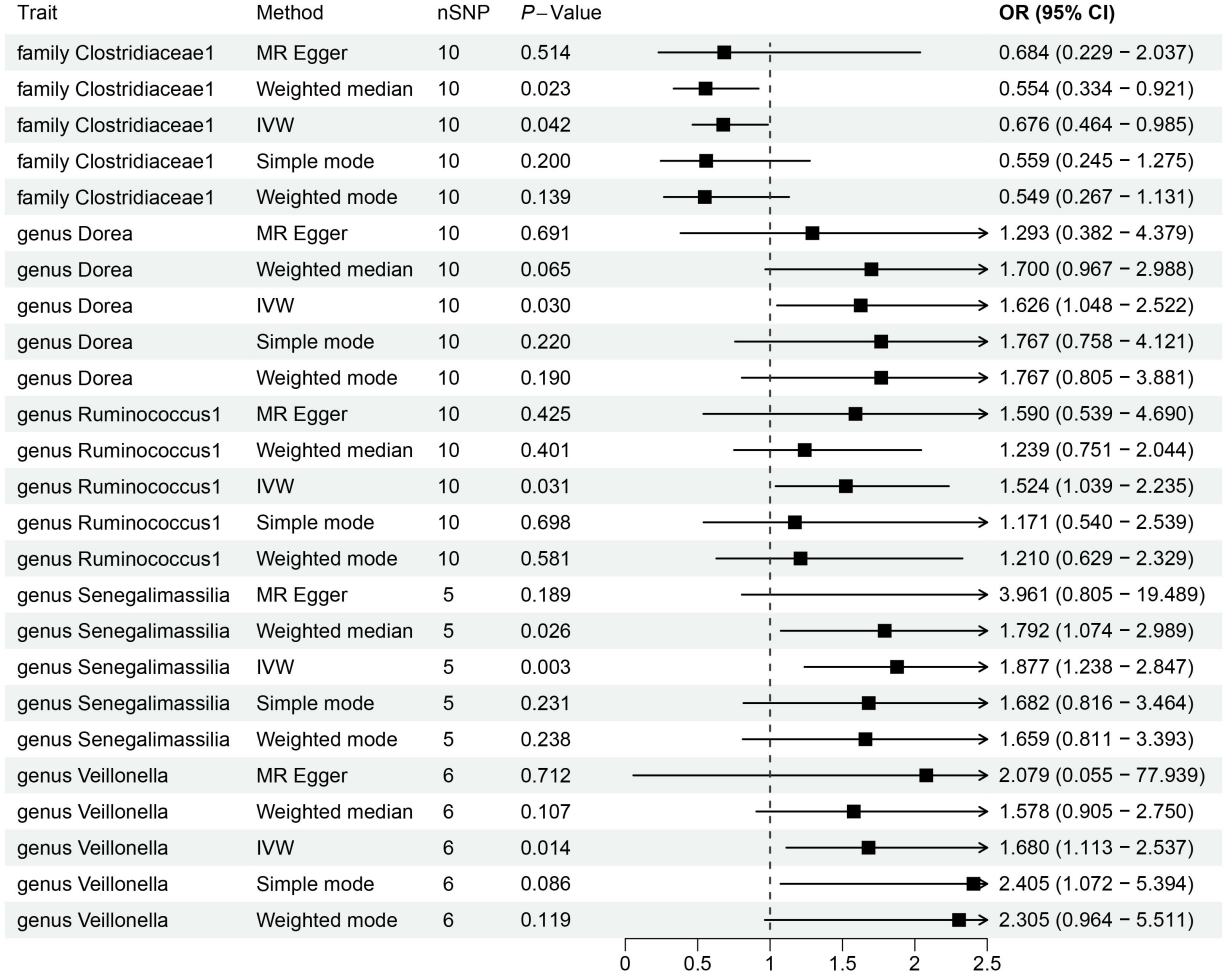
Forestplot showing significant causal effects between gut microbiota and OL.

**Figure 3:**
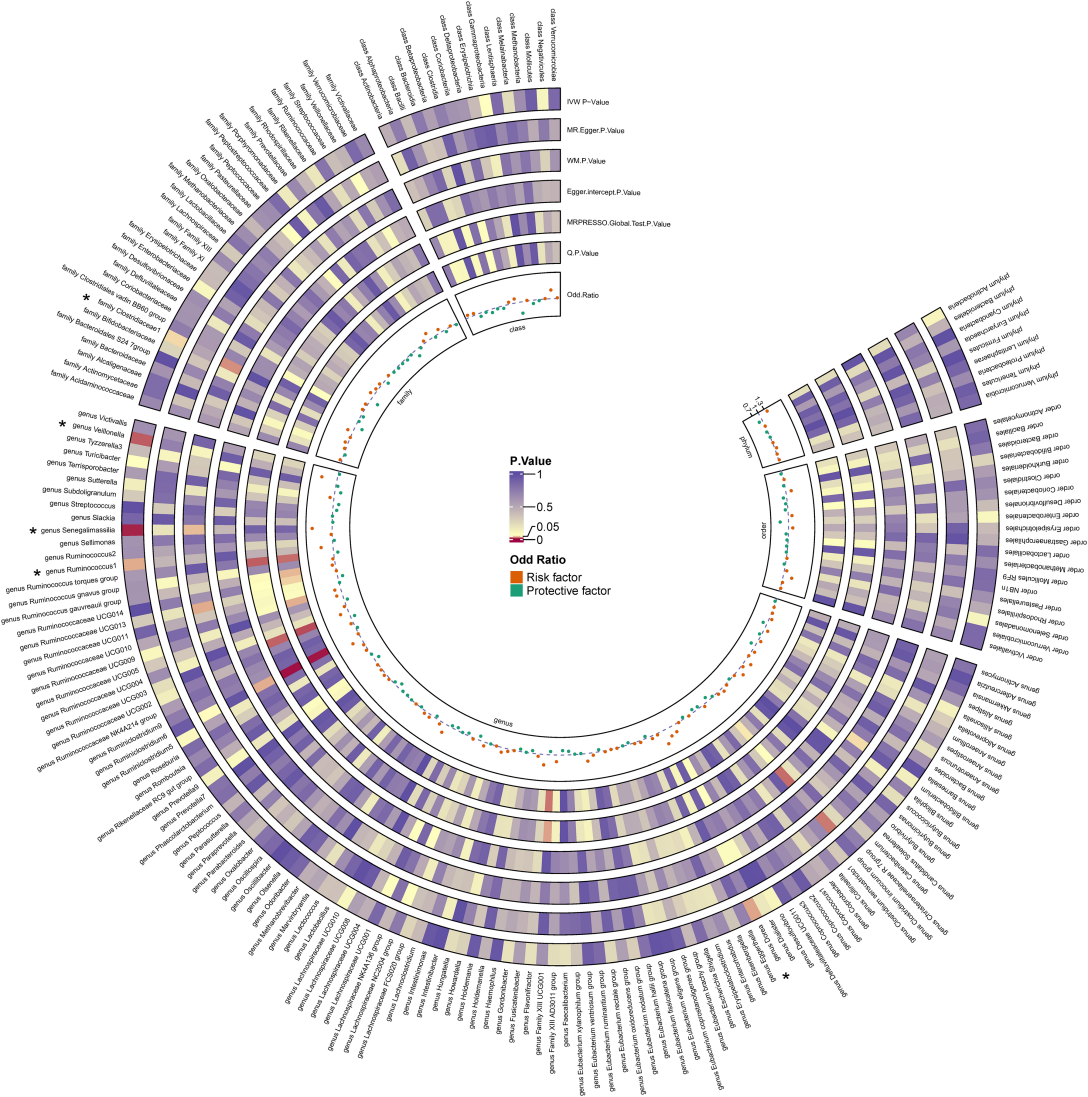
Heatmap for causality of gut microbiota on OL. Causality of 211 gut bacterial taxa on OL. Results that reached statistical significance in the IVW analysis are marked with an asterisk (*) and represent a P.value of less than 0.05.

**Figure 4:**
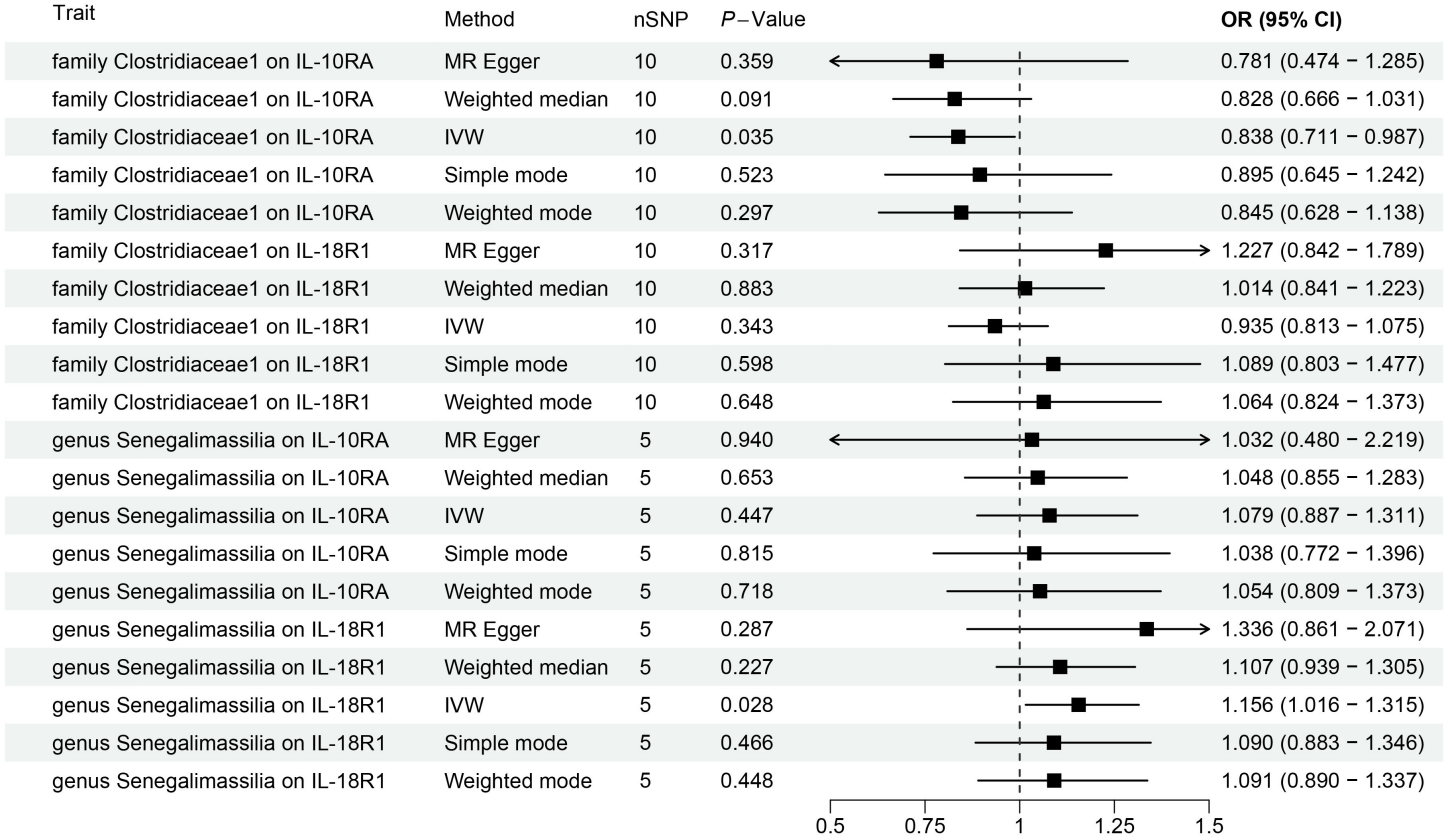
Forest plots for Mendelian randomization results of causal effects between gut microbiotas and 2 inflammation-related proteins (IL-10RA and IL18-R1).

By utilizing Mendelian randomization, we delved into the relationship between specific gut microbiota and OL (**Figure 1**). The preliminary findings indicate that 5 out of 211 gut bacterial taxa are causally associated with oral leukoplakia (**Figures 2**, **3**). The results of IVW analysis of these 5 bacterial taxa are shown below: *family Clostridiaceae1*(*P*= 0.042;odds ratio [OR] 95% confidence interval [CI]= 0.676 [0.464 − 0.985]), *genus Dorea* (*P*= 0.030; OR[95%CI]= 1.626 [1.048 − 2.522]), *genus Ruminococcus1*(*P*= 0.031; OR=95%[CI]= 1.524 [1.039 − 2.235]), genus Senegalimassilia(*P*= 0.003; OR[95%CI]= 1.877 [1.238 − 2.847]), *genus Veillonella*(*P*= 0.014; OR[95%CI]= 1.680 [1.113 − 2.537]). The *family Clostridiaceae1* was negatively associated with the risk of oral leukoplakia development, whereas the other taxa were positively associated with the risk of oral leukoplakia development, suggesting that their abundance in the gut microbiota may contribute to the development of OL. The **Figure 4**; **Supplementary Materials** provide detailed information on MR analysis of gut microbiota and oral leukoplakia (**Supplementary Table S1**). Heterogeneity test of gut microbiota and oral microbiota showed that all p-values exceeded 0.05, and no evidence of horizontal pleiotropy was found using the MR Egger method (**Supplementary Table S4**). The sensitive analysis further validated these findings (**Supplementary Tables S2–S4**).

For the causal relationship between the gut microbiota and oral leukoplakia mentioned above, we conducted reverse MR (**Supplementary Table S5**), and found a causal relationship between oral leukoplakia and *family Bacteroidales S24 7group*(*P*=0.0048), *family Christensenellacea* (*P*=0.0414), *genus Intestinibacte* (*P*=0.0479) and *genus Lachnospiraceae* (*P*=0.0298).

### 3.2 The causal effect of inflammation-related proteins on OL

**Figure 5:**
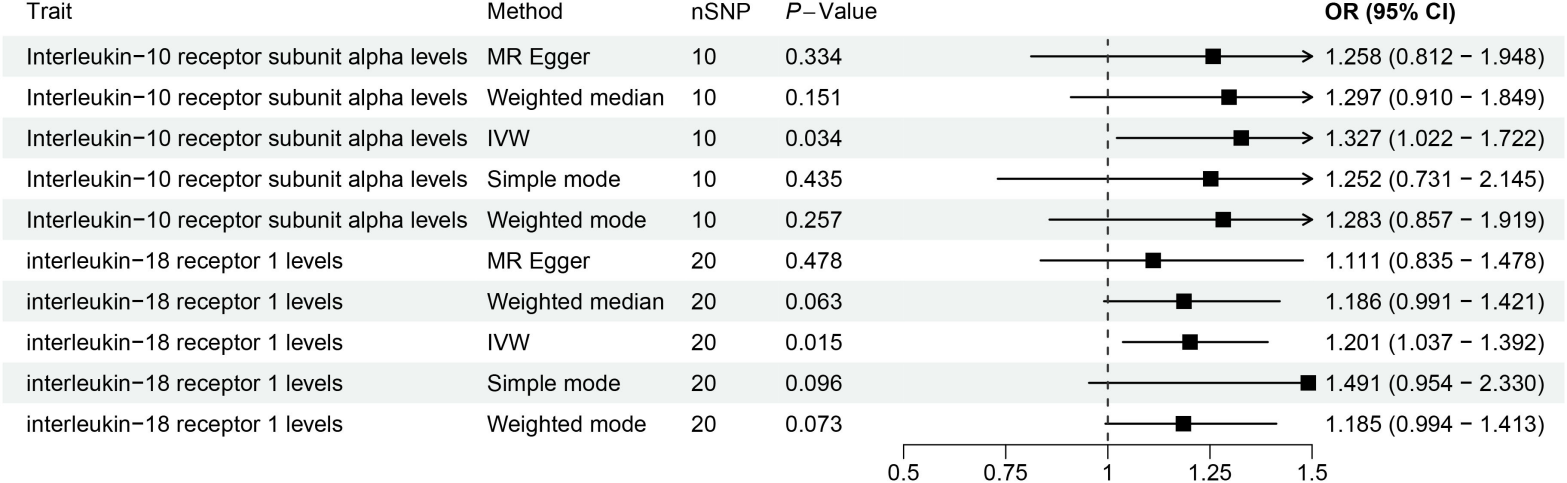
The causal association results of causal effects between 2 inflammation-related proteins (IL-10RA and IL18-R1) and OL.

This study revealed a causal relationship between 91 inflammation-related proteins and oral leukoplakia, with only 2 inflammation-related proteins exhibiting a potential causal relationship with oral leukoplakia (**Supplementary Table S6**; **Figure 5**). IVW analysis shows that, IL-10RA (OR[95%CI]= 1.327[1.022-1.722], *P*= 0.034) and IL18-R1 (OR[95%CI]=1.201[1.037-1.392], *P*= 0.015) are risk factors for oral leukoplakia. Sensitivity analysis was used to verify that these results have no heterogeneity or level pleiotropy (**Supplementary Tables S7–S9**).

### 3.3 Mendelian randomization analysis of gut microbiota and inflammation-related proteins

We conducted MR analysis on the gut microbiota and 91 inflammation-related proteins to further elucidate the role of these inflammation-related proteins in the relationship between gut microbiota and oral leukoplakia (**Supplementary Table S11**; **Figure 4**). The IVW analysis results showed a causal relationship between *family Clostridiaceae1* and IL-10RA (OR[95%CI]=0.84[0.71, 0.99], *P*=0.035). There is a causal relationship between *genus Senegalimassilia* and IL18-R1 (OR[95%CI]=1.16 [1.02, 1.31], *P*=0.028), and no significant association was observed between any other bacterial taxa and inflammation-related proteins.

### 3.4 Mediation analysis results of gut microbiota, inflammation-related proteins and OL

**Figure 6:**
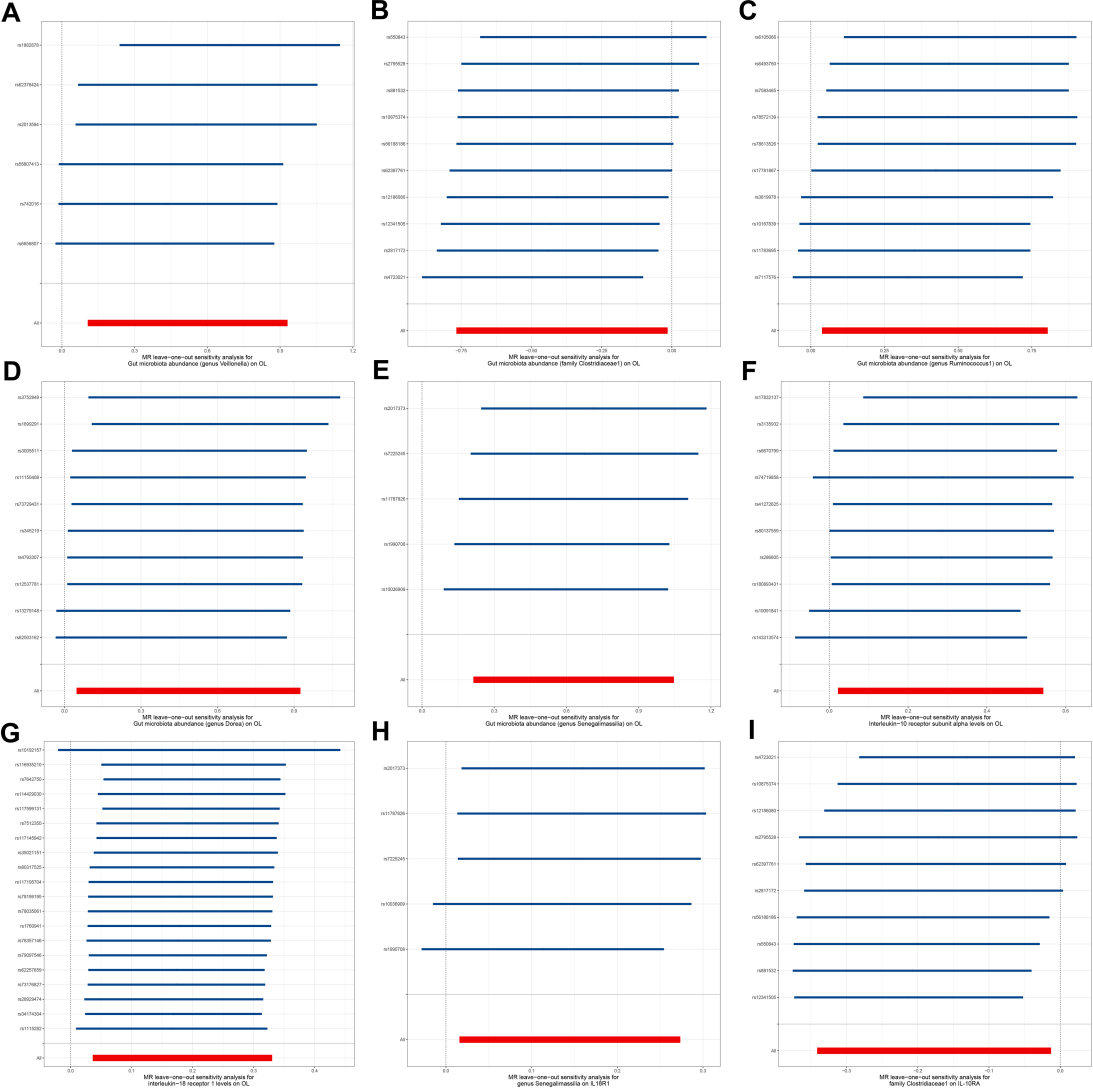
Leave-one-out plot. **(A)** Leave-one-out plot of *genus Veillonella* and OL, **(B)** Leave-one-out plot of *family Clostridiaceae1* and OL; **(C)** Leave-one-out plot of *genus Ruminococcus1* and OL; **(D)** Leave-one-out plot of *genus Dorea* and OL; **(E)** Leave-one-out plot of *genus Senegalimassilia* and OL; **(F)** Leave-one-out plot of IL-10RA and OL; **(G)** Leave-one-out plot of IL18-R1 and OL; **(H)** Leave-one-out plot of *genus Senegalimassilia* and IL18-R1; **(I)** Leave-one-out plot of *family Clostridiaceae1* and IL-10RA.

To explore the potential mechanisms underlying the occurrence and development of oral leukoplakia, we conducted a mediation analysis to determine the causal pathway mediated by inflammation-related proteins from the gut microbiota to oral leukoplakia. This analysis focuses on the previously identified gut microbiota and inflammation-related proteins associated with oral leukoplakia in two-sample MR. In this study, both gut microbiota and inflammation-related proteins have a causal effect on OL inflammation-related proteins seem to play a mediating role in the pathways of gut microbiota and oral leukoplakia. Mediation analysis shows that the indirect effect of *gens Senegalimassilia* on oral leukoplakia through IL18-R1 is β= 0.026 (95%CI: 0.001-0.063), with a mediation ratio of 4.21%. Additionally, we also conducted a leave-one-out analysis, which confirmed the stability of our findings. The leave-one-out plot is shown in **Figure 6**, with heterogeneity results and pleiotropy analysis results provided in **Supplementary Tables S2–S4**. The leave-one-out analysis helps assess the stability and reliability of the results in Mendelian randomization study by showing how sensitive the overall findings are to the inclusion or exclusion of individual genetic variants. This indicates that IL18-R1 play a mediating role in the pathway between the gut microbiota *genus Senegalimasilia* and oral leukoplakia.

## 4 Discussion

Few studies have explored the relationship between gut microbiota and oral leukoplakia. In this study, we used MR studies to explore the potential causal relationship between gut microbiota and oral leukoplakia. We analyzed the relationship between the abundance of 211 common gut microbiota and oral leukoplakia. The results indicate that certain specific gut microbiota are risk factors, while others are protective factors for oral leukoplakia. High abundance of *family Clostridiaceae1* can reduce the risk of oral leukoplakia, while high abundance of *genus Dorea*, *genus Ruminococcus1*, *genus Senegalimasilia*, and *genus Veillonella* may increase the risk of oral leukoplakia. The available studies usually consider *genus Dorea* as a harmful microorganism. Chiu et al. showed a significant correlation between *genus Dorea* and allergic rhinitis (39). Nitsan et al. found that luminal proportions of *genus Dorea* were significantly higher in fecal samples from patients with irritable bowel syndrome (IBS) than in healthy controls (40). *Genus Ruminococcus1* is an anaerobic bacterium that, as a gram-positive bacterium, plays different roles in various diseases. Although *genus Ruminococcus1* can ferment complex sugars and produce short chain fatty acids (SCFAs), SCFAs have been shown to be beneficial in reducing the risk of certain cardiovascular metabolic diseases by reducing visceral fat. The research results of Zhang et al. indicate that *genus Ruminococcus1* has a protective effect on blood vessels and can reduce the risk of Intracranial aneurysm and aneurysm subarachnoid hemorrage (41). While in some digestive system diseases, spondylitis and asthma, the abundance of *genus Ruminococcus1* significantly increases (42). The results of Li et al. found that *genus Ruminococcus1* may act synergistically with other bacteria to promote an increase in lipopolysaccharide and exacerbate intestinal barrier damage (43).*Genus Senegalimassilia* are nonmotile, Gram-stain-positive, anaerobic and coccobacilli. The *genus Senegalimassilia* is positively associated with potential cardiovascular disease risk biomarkers (44), Wang et al. showed that *genus Senegalmassilia* was positively associated with blood pressure (45), Hypertension can damage endothelial cells, affect vascular dilation and blood flow, and thus affect the health status of the oral mucosa. Previous studies have reported an increase in the abundance of *genus Veillonella* in patients with oral leukoplakia, which is consistent with our observations (46). This indicates a potential connection between specific gut microbiota and the development of oral leukoplakia.

The human gut microbiota contains over 10 trillion microbes that reside in the human intestine. Similarly, the oral microbiota, the second-largest microbiota in the body, consists of over 700 bacterial species (12). Given the physical connection between the oral and gastrointestinal mucosa, and the ingestion of saliva daily, there is a significant interplay between oral and gut microbiota (47). Oral bacteria can influence the gut microbiota through various routes, including the enteral route via saliva (48), the hematogenous route via bloodstream spread (49), and immune cell migration (50). These interactions underscore the close relationship between oral and gut microbiota, with the former potentially impacting gut health and systemic diseases (16). Recent studies have highlighted a complex relationship between OL and alterations in the oral microbiota. While results vary across investigations, they generally indicate a shift in microbial communities associated with OL. Alfa-diversity studies have reported inconsistent findings, ranging from increased bacterial richness to depletion or no difference between OL and healthy controls. However, beta-diversity has consistently shown differences between these groups, though some results are complicated by variations in subsites or subgroups (51, 52). Several studies have identified specific bacteria associated with OL. Ganly et al. found an enrichment of periodontal pathogens like *Fusobacterium*, *Prevotella*, and *Alloprevotella* in OL, along with a depletion of commensal *Streptococcus* (46). Amer et al. reported decreased levels of *Firmicutes* and an increase in *Fusobacterium nucleatum*, *Campylobacter* spp., and *Rothia mucilaginosa* in OL, while healthy mucosa was enriched in *Streptococcus mitis* and *Gemella haemolysans* (51). Similarly, Decsi et al. observed increased *Fusobacterium nucleatum* and decreased *Streptococcus mitis* in OL (14). In addition to bacterial shifts, fungal colonization, particularly by *Candida* species, has been noted in OL. Amer et al. found *Candida* in 35% of OL samples, significantly higher than in healthy controls, with specific clusters showing a strong association with *Candida* (51). The role of fungi in oral premalignant disorders is ambivalent, with some species like *Malassezia* and *Schizophyllum* showing anti-carcinogenic potential, while *Candida* species have been linked to carcinogenesis. The potential interaction between bacteria, fungi, and viruses in OL pathogenesis remains largely unexplored and warrants further investigation. In conclusion, OL is associated with significant changes in the oral microbiota, characterized by a decrease in commensals like *Streptococci* and an increase in anaerobes such as *Fusobacterium nucleatum* and *Porphyromonas gingivalis*. These microbial shifts may serve as markers for the progression of OL to oral squamous cell carcinoma (OSCC) (53). While there is indeed a substantial body of literature on the oral microbiome’s role in oral cancer and OL, the gut microbiome’s potential influence remains underexplored. Our study aims to contribute novel insights by investigating whether gut microbiota can have a systemic effect on the pathogenesis of OL, thus broadening the understanding of microbial contributions beyond the oral cavity. This approach may reveal new therapeutic targets or preventive strategies by modulating the gut microbiome, which could complement existing oral microbiome-focused interventions.

This study determined the impact of gut microbiota on oral leukoplakia by their relative abundance expression. However, the exact mechanism by which gut microbiota leads to oral leukoplakia has not been determined. We assume that inflammation-related proteins may be a mediating factor between the gut microbiota and oral leukoplakia. According to MR analysis, we found that IL-10RA and IL18-R1 significantly increased the risk of oral leukoplakia. Our study provides genetic evidence that IL18-R1 plays a mediating role in the effect of *genus Senegalimasilia* on OL.

Our MR study provides compelling genetic evidence that 2 inflammation-related proteins (IL-10RA and IL18-R1) are associated with the etiology of oral leukoplakia. Notably, IL18 was identified as a risk factor for patients with OL. In a previous report, Zhang et al. used an enzyme-linked immunosorbent assay to screen serum and salivary concentrations of IL-18 and showed that elevated serum and salivary IL-18 correlated with disease severity in patients with OL, and these findings could be considered the predictive or prognostic value of elevated IL-18 for OL (21). The key pro-inflammatory cytokine IL-18 plays an important role in the control of innate and adaptive immunity. The receptor IL18-R1 belongs to the interleukin-1 receptor family and is expressed in Th1 cells. After binding with IL18, IL-18 triggers the recruitment of IL18RAP, thereby initiating signal transduction (54, 55). IL-18 is secreted by antigen-presenting cells, stimulates IFN - γ synthesis, and participates in the synergistic activation of interleukin-12 to promote Th1 mediated immune responses. IL18-R1 plays an important role in cancer progression. For example, IL18R1 was over-expressed in breast cancer tissues compared with nearby non-cancerous tissues (56). In addition, the expression level of IL18-R1 is positively correlated with disease-free survival (DFS) in patients with triple negative breast cancer (57), This indicates that it is useful as a prognostic marker for this malignant tumor. Human IL-10R is a heterotetramer composed of receptor chains IL-10RA and IL-10RB, belonging to class II cytokine receptors (58), expressed on innate immune cells and adaptive immune cells. The IL-10RA chain plays a dominant role in mediating high affinity ligand binding and signal transduction. The interaction between IL-10 and IL-10R complex stabilizes the dimerization of two IL-10R subunits, activating Janus kinase 1 and tyrosine kinase 2, and catalyzing phosphorylation of themselves, and induction of intercellular signal transduction. Usually, The expression level of IL-10RA is related to the intensity of IL-10’s effect on immune cells (59). Research has shown that due to its immunosuppressive function, the presence of Tregs in the subcutaneous matrix of vitiligo is a potential indicator of cancer progression (60). The expression of higher quantities of Treg related proteins (including IL-10) may have potential predictive effects in malignant transformation (61). Previous studies have shown that mutations in the first splicing site of IL10RA lead to inflammatory bowel disease (IBD) in infants. In addition, the phenotypes of IL10RA polymorphism include severe arthritis and Very early onset Ulcerative Colitis (62). Our research findings indicate that IL-10RA and IL-18R1 are risk factors for precancerous lesions associated with oral leukoplakia, thereby strengthening their association with oral leukoplakia and providing new insights into their role in the pathogenesis of oral leukoplakia.

This is the first large-scale MR analysis of the causal relationship between gut microbiota, inflammation-related proteins and OL. The exploration of the relationship between gut microbiota, inflammation-related proteins and OL not only elucidates potential prevention and treatment strategies, but also helps to deepen understanding of the pathogenesis of OL. In addition, the identification of specific gut microbiota associated with the risk of OL provides promising targets for future interventions and treatment methods aimed at regulating gut microbiota to reduce the risk of OL. But our research has some limitations. Firstly, since the samples come from European ancestry, the applicability of our research results is limited, which may not accurately reflect the impact of genetic and lifestyle diversity on the gut microbiota of different populations. Secondly, the association between certain microbial communities we have observed and the risk of oral leukoplakia is preliminary. Considering the complexity of the interactions between gut microbiota, further research should be conducted on their mechanisms of action. Thirdly, we highlight MR method’s ability to infer causal relationships using genetic variants as instrumental variables, thereby reducing confounding factors. However, we also acknowledge the limitations, such as the potential for pleiotropy and the assumption that the genetic variants only influence the outcome through the exposure of interest. By addressing these points, we aim to provide a balanced perspective on the use of MR in our study. Finally, although we investigated the mediating role of 91 inflammation-related proteins between different gut microbiota abundance and oral leukoplakia, the mechanism by which gut microbiota affects the occurrence and development of oral leukoplakia through inflammation-related proteins remains to be studied.

## 5 Conclusion

This study provides new evidence for the causal relationship between gut microbiota, inflammation and OL. Our MR study suggests that inflammation-related proteins mediate the pathway of gut microbiota leading to OL. These findings provide new insights into the pathogenesis of OL, indicating their potential significance in preventing and treating OL.

## Data Availability

The original contributions presented in the study are included in the article/**Supplementary Material**. Further inquiries can be directed to the corresponding authors.
