# Supplementary material for: Effect of the gut microbiome and inflammation-related proteins on oral leukoplakia: a Mendelian randomization study and mediation analysis
Source: Front Oncol. 2024 Sep 25;14:1443123. doi: 10.3389/fonc.2024.1443123 (PMC11461448; doi:10.3389/fonc.2024.1443123)
Supplement: Supplementary file 2 [file DataSheet2.docx]

Supplementary Material

# Supplementary Figures


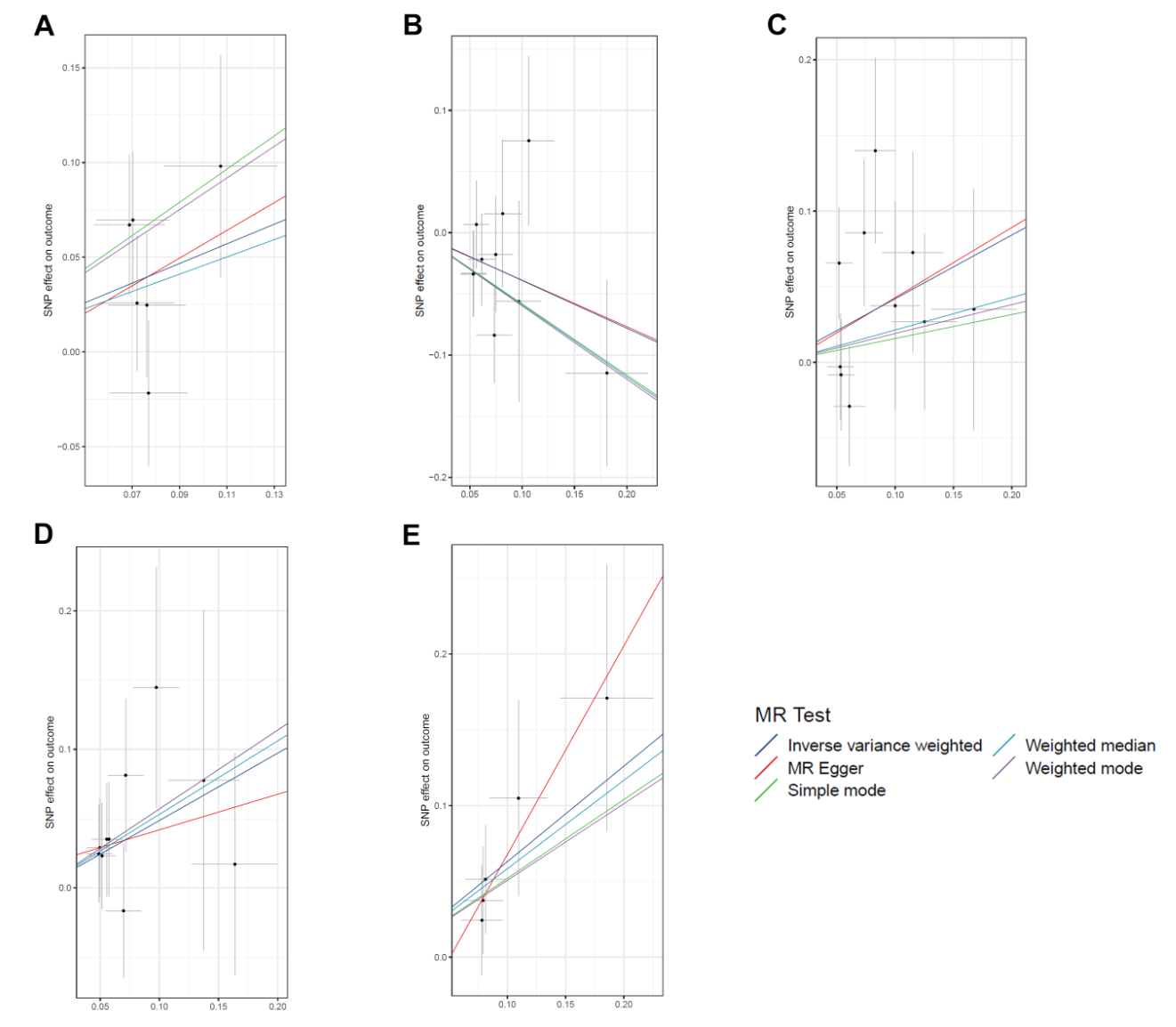


**Figure S1 Scatter plots for the causal associations between gut microbiotas on OL.** Each black point represents the SNP effect sizes on the exposure (horizontal axis) and outcome (vertical axis). (A) Scatter plot of genus Veillonella and OL, (B) Scatter plot of family Clostridiaceae1 and OL; (C) Scatter plot of genus Ruminococcus1 and OL; (D) Scatter plot of genus Dorea and OL; (E) Scatter plot of genus Senegalimassilia and OL
